# Metoprolol blunts the time-dependent progression of infarct size

**DOI:** 10.1007/s00395-020-0812-4

**Published:** 2020-08-03

**Authors:** Manuel Lobo-Gonzalez, Carlos Galán-Arriola, Xavier Rossello, Maribel González‐Del‐Hoyo, Jean Paul Vilchez, María I. Higuero-Verdejo, Jose M. García-Ruiz, Gonzalo J. López-Martín, Javier Sánchez-González, Eduardo Oliver, Gonzalo Pizarro, Valentin Fuster, Borja Ibanez

**Affiliations:** 1 Translational Laboratory for Cardiovascular Imaging and Therapy, Centro Nacional de Investigaciones Cardiovasculares (CNIC), Madrid, Spain; 2 Cardiology Department, IIS-Fundación Jiménez Díaz University Hospital, Madrid, Spain; 3 Centro de Investigación Biomédica en Red en Enfermedades Cardiovasculares (CIBERCV), Madrid, Spain; 4 Cardiology Department, IdISBa-Hospital Universitari Son Espases, Palma de Mallorca, Spain; 5 Cardiology Department, University Hospital of Tarragona Joan XXIII, IISPV, Rovira i Virgili University, Tarragona, Spain; 6 Complejo Hospitalario Ruber Juan Bravo, Madrid, Spain; 7 Hospital de Cabueñes, Gijón, Spain; 8 Philips Healthcare, Madrid, Spain; 9 Icahn School of Medicine at Mount Sinai, New York, NY USA

**Keywords:** Acute myocardial infarction, Ischemia–reperfusion injury, Metoprolol, Early reperfusion

## Abstract

Early metoprolol administration protects against myocardial ischemia–reperfusion injury, but its effect on infarct size progression (ischemic injury) is unknown. Eight groups of pigs (total *n* = 122) underwent coronary artery occlusion of varying duration (20, 25, 30, 35, 40, 45, 50, or 60 min) followed by reperfusion. In each group, pigs were randomized to i.v. metoprolol (0.75 mg/kg) or vehicle (saline) 20 min after ischemia onset. The primary outcome measure was infarct size (IS) on day7 cardiac magnetic resonance (CMR) normalized to area at risk (AAR, measured by perfusion computed tomography [CT] during ischemia). Metoprolol treatment reduced overall mortality (10% vs 26%, *p* = 0.03) and the incidence and number of primary ventricular fibrillations during infarct induction. In controls, IS after 20-min ischemia was ≈ 5% of the area AAR. Thereafter, IS progressed exponentially, occupying almost all the AAR after 35 min of ischemia. Metoprolol injection significantly reduced the slope of IS progression (*p* = 0.004 for final IS). Head-to-head comparison (metoprolol treated vs vehicle treated) showed statistically significant reductions in IS at 30, 35, 40, and 50-min reperfusion. At 60-min reperfusion, IS was 100% of AAR in both groups. Despite more prolonged ischemia, metoprolol-treated pigs reperfused at 50 min had smaller infarcts than control pigs undergoing ischemia for 40 or 45 min and similar-sized infarcts to those undergoing 35-min ischemia. Day-45 LVEF was higher in metoprolol-treated vs vehicle-treated pigs (41.6% vs 36.5%, *p* = 0.008). In summary, metoprolol administration early during ischemia attenuates IS progression and reduces the incidence of primary ventricular fibrillation. These data identify metoprolol as an intervention ideally suited to the treatment of STEMI patients identified early in the course of infarction and requiring long transport times before primary angioplasty.

## Introduction

Despite major improvements in reperfusion strategies and coadjuvant therapies, myocardial infarction (MI) remains a leading cause of mortality and morbidity worldwide [21, 30]. Infarct size (IS) is a major determinant of prognosis in MI survivors; therefore, interventions able to limit it are needed. Since the extent of irreversible injury progresses in a time-dependent manner, early blood flow restoration (reperfusion) is associated with lower IS, and this translates into better long-term outcomes [3, 33]. This notion is the basis of the widely applied “time is muscle” principle [18]. If provided early, the best reperfusion strategy for patients presenting with a ST-elevation MI (STEMI) is primary percutaneous coronary intervention (PPCI). PPCI stops ischemic injury progression immediately and performs better than fibrinolysis in achieving adequate tissue perfusion and limiting microvascular injury. Unfortunately, PPCI is often not an immediate option, and reperfusion is delayed during patient transfer to a PCI facility. Current guidelines recommend PPCI if the anticipated time from STEMI diagnosis to wire crossing is ≤ 120 min [21]. If a longer transfer time is predicted, the recommended reperfusion strategy is systemic fibrinolysis. However, fibrinolysis results in complete reperfusion in only ~ 50% of treated patients and exposes patients to potentially serious bleeding events, such as intracranial hemorrhage [21]. Interventions to delay the progression of ischemic damage could theoretically extend the 120 min window for selecting PPCI over fibrinolysis, allowing more patients to benefit from the best reperfusion strategy.

Intravenous (i.v.) administration of metoprolol during ongoing ischemia is associated with smaller infarcts, both in experimental models [6, 7, 23] and in a recent clinical trial [22, 27]. In experimental and clinical settings, the cardioprotective effect of i.v. metoprolol is dependent on the timing of administration; when injected very close to reperfusion, metoprolol has no infarct-limiting effect [7]. This timing-dependent cardioprotective effect might explain the neutral effects reported in another recent clinical trial [29]. The fact that i.v. metoprolol reduced IS only when injected long before reperfusion suggests that it might slow the rate of ischemic death (i.e. infarction progression).

To address this question, we studied the trajectories of IS progression in the presence or absence of i.v. metoprolol in a pig model of STEMI. To mimic the clinical scenario, i.v. metoprolol or vehicle was injected 20 min after coronary artery occlusion, and pigs were reperfused at different times, from immediately after injection to 40 min later (i.e. 60 min after ischemia onset). All pigs underwent cardiac magnetic resonance (CMR) imaging examinations at 7 and 45 days after STEMI induction.

## Methods

The study was conducted at *Centro Nacional de Investigaciones Cardiovasculares* (CNIC) facilities and approved by Institutional and Regional Animal Research Committees. All animal procedures conformed to EU Directive 2010/63EU and Recommendation 2007/526/EC regarding the protection of animals used for experimental and other scientific purposes.

### Study design

**Fig. 1:**
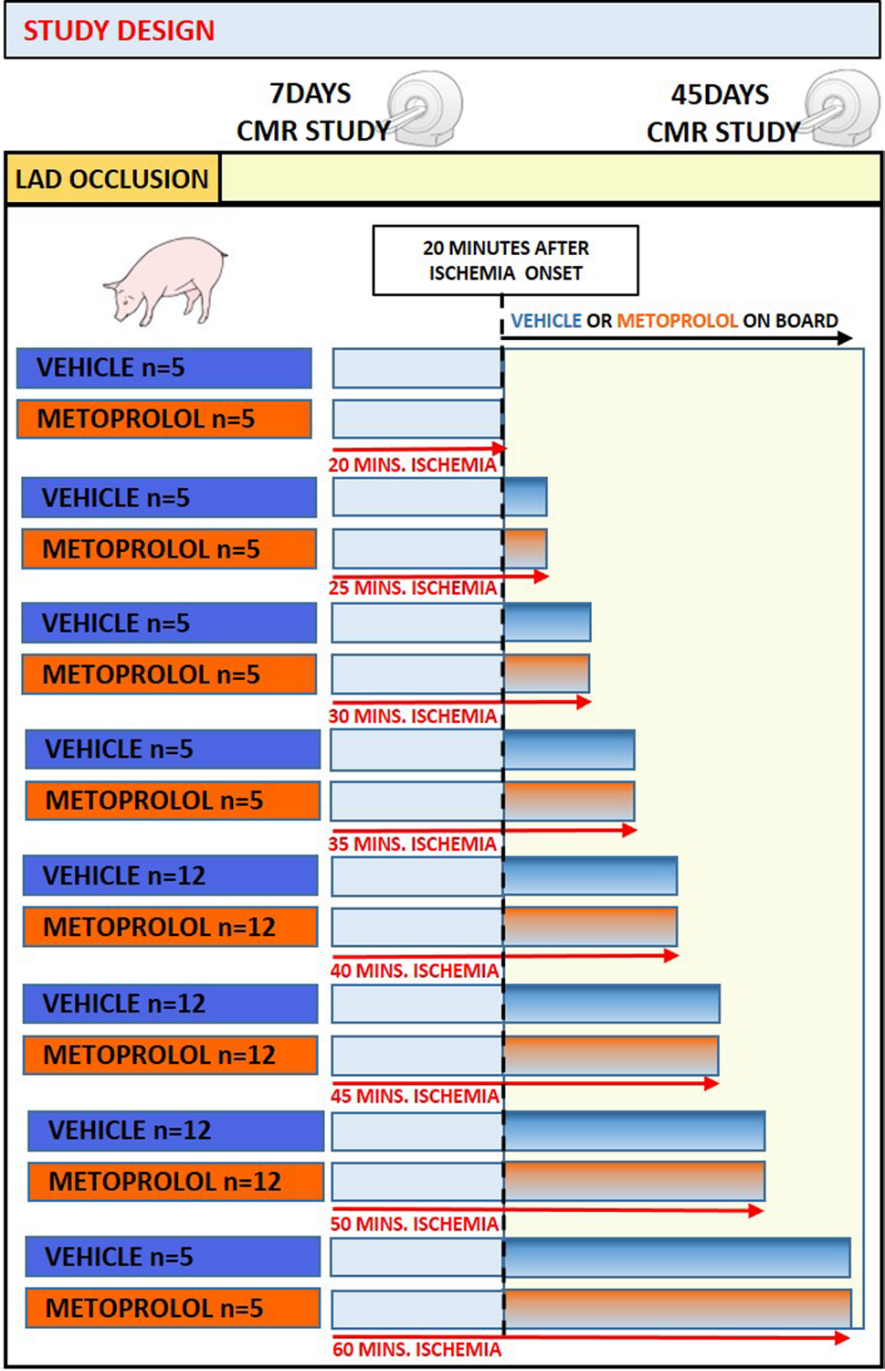
Study design. Large white pigs (30–35 kg) were allocated to eight periods of ischemia by left anterior descending occlusion for 20 (*n* = 10), 25 (*n* = 10), 30 (*n* = 10), 35 (*n* = 10), 40 (*n* = 24), 45 (*n* = 24), 50 (*n* = 24), or 60 (*n* = 10) min, followed by reperfusion. Animals were 1:1 randomized to receive either intravenous metoprolol (0.75 mg/kg) or saline 20 min after ischemia onset. MDCT scans were performed during the course of ischemia. Cardiac magnetic resonance scans were performed 7 days after reperfusion, and 45 days after reperfusion

The study design is presented in Fig. 1. The study was performed in 3-month-old male large white pigs weighing 30–35 kg following state-of-the art methodologies [1, 17, 25]. Eight groups of pigs were scheduled to undergo ischemia for differing durations (20 min, 25 min, 30 min, 35 min, 40 min, 45 min, 50 min, or 60 min). Ischemia was induced by inflating an angioplasty balloon in the mid-portion of the left anterior descending (LAD) coronary artery. At the end of the pre-specified ischemia duration, animals were reperfused (balloon deflation) and recovered from anesthesia. All pigs underwent contrast-enhanced multidetector computed tomography (MDCT) during coronary occlusion to depict the area at risk (AAR) [3]. All pigs were scheduled to undergo two CMR exams, at 7 and 45 days after infarction induction. Animals in each ischemia time group were randomly allocated to i.v. metoprolol (0.75 mg/kg of 1 mg/ml Beloken®, Casen Recordati, Italy) [7] or i.v. vehicle (0.75 ml/kg saline). All metoprolol and vehicle injections were at 20 min after ischemia onset. Drug or vehicle was administered always at 20 min after the ischemia onset, in order to mimic the normal delay between symptoms and medical assistance in clinical arena. Operators were blinded to treatment allocation. The sample size of pigs scheduled to 40 min, 45 min, and 50-min I/R was *N* = 12 per group, whereas sample size for the 20-min, 25-min, 30-min, 35-min, and 60-min I/R groups was *N* = 5 per group. Dead animals were not replaced. All animals (including those allocated to vehicle during ischemia) received 50 mg daily oral metoprolol throughout the study, starting the day after I/R induction. Clopidogrel (150 mg) was administered immediately after reperfusion, followed by 75 mg daily for two additional days.

The pre-specified primary endpoint IS [extent of late gadolinium enhancement (LGE) normalized to AAR] on day 7 CMR. Secondary outcomes were microvascular obstruction (MVO) on day 7 CMR as well as left ventricular (LV) volumes and LV systolic function (LV ejection fraction, LVEF) on day 45. For comparative purposes, hemodynamic parameters (systolic and diastolic blood pressure) before ischemia onset and upon reperfusion, incidence and timing of ventricular fibrillation (VF) events, and mortality were recorded in all animals throughout the study.

### Pig model of STEMI (ischemia–reperfusion (I/R) protocol)

The MI-induction protocol is detailed elsewhere [5, 32]. In brief, sedation was induced by intramuscular injection of ketamine (20 mg/kg), xylazine (2 mg/kg), and midazolam (0.5 mg/kg) and maintained by continuous intravenous infusion of ketamine (2 mg/kg/h), xylazine (0.2 mg/kg/h), and midazolam (0.2 mg/kg/h). The analgesic buprenorphine (0.03 mg/kg) was administered by intramuscular injection immediately before the procedure. Animals were intubated and received mechanical ventilatory support with mandatory synchronized intermittent volume-control ventilation (fraction of inspired O_2_, 28%). Central venous and arterial lines were inserted, and a single bolus of unfractionated heparin (300 IU/kg) was administered at the onset of the instrumentation. During the procedure, animals were continuously infused with amiodarone (300 mg/h). Amiodarone was initiated immediately after coronary artery occlusion. Through the femoral arterial sheath, a guiding catheter was placed in the left main coronary artery. A coronary wire was placed distal in the LAD, and an angioplasty balloon was inflated in the mid-LAD, occluding the LAD immediately distal to the origin of the first diagonal branch. Balloon location, maintenance of inflation, and post-reperfusion patency were monitored by contrast angiography. In cases of VF, non-synchronized biphasic defibrillations were applied until sinus rhythm was restored.

### Arterial enhanced MDCT protocol and AAR quantification

After coronary artery occlusion, pigs were moved to the MDCT suite. In all cases, pigs were back in the catheterization laboratory within 15 min of ischemia onset. Arterial phase MDCT studies were performed in a 64-slice CT scanner (Brilliance CT 64; Philips Healthcare, Cleveland, OH) after intravenous administration of iodinated contrast medium. MDCT images were evaluated with dedicated software (MR Extended Work Space 2.6; Philips Healthcare, Best, The Netherlands) by two observers blinded to ischemia duration protocol and treatment allocation. Short-axis orientation images were obtained from volumetric CT images by multiplanar reconstruction. The region negative for contrast enhancement corresponds to the territory supplied by the occluded vessel (AAR) and was identified based on contrast enhancement differences vs the remote myocardium. The AAR was manually delineated and expressed as a percentage of LV area.

### CMR protocol and analysis

Cine and contrast-enhanced CMR studies were performed 7 days and 45 days after MI induction. CMR examinations were conducted using a Philips 3-T Achieva Tx whole body scanner (Philips Healthcare, Best, The Netherlands) equipped with a 32-element phased-array cardiac coil. The imaging protocol included a standard segmented cine steady-state free-precession sequence to provide high-quality anatomic references and assessment of LV volumes, LV mass, and LVEF, as well as a T1-weighted inversion recovery turbo field echo sequence acquired 10–15 min after the administration of gadolinium contrast to assess IS (extent of LGE) and MVO (dark regions within the LGE region). CMR images were evaluated with dedicated software (MR Extended Work Space 2.6; QMassMR 7.6; Medis, Leiden, The Netherlands) by 2 observers experienced in CMR analysis and blinded to group allocation.

### Statistical analysis

Sample size was calculated according to previous results, for a power 80%, significance level of 5% and expected mortality of 30%.

Categorical variables are expressed as percentages and were compared by the chi-square test (or the Fisher exact test when appropriate). Normal distribution of each data subset was checked using graphical methods and a Shapiro–Wilk test. Continuous variables are expressed as mean ± standard deviation if normally distributed and otherwise as median (interquartile range [IQR]). Between-group comparisons were made by parametric methods (nonpaired Student’s *t* test) or nonparametric methods (Mann–Whitney *U* test) as appropriate. Differences were considered statistically significant at *p* < 0.05 (two tailed).

Cumulative mortality was calculated using the Kaplan–Meier method. The difference in survival estimates across dipstick categories was assessed by the log-rank test.

Slopes of myocardial damage over ischemia time in the vehicle and metoprolol groups were adjusted using an asymmetric sigmoidal model (least squares regression).

All statistical analyses were performed with Stata v15.1 (StataCorp, College Station, TX). Graphs were generated with GraphPad-Prism v7.0 (GraphPad Software, Inc, La Jolla, CA).

## Results

**Fig. 2:**
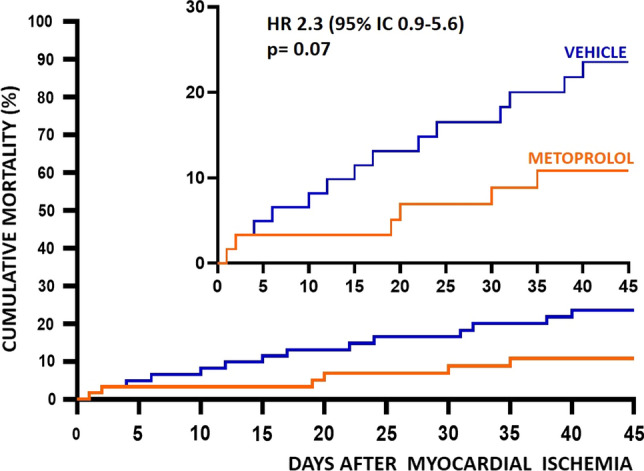
Kaplan–Meier survival curves in pigs receiving metoprolol or vehicle during ischemia

**Fig. 3:**
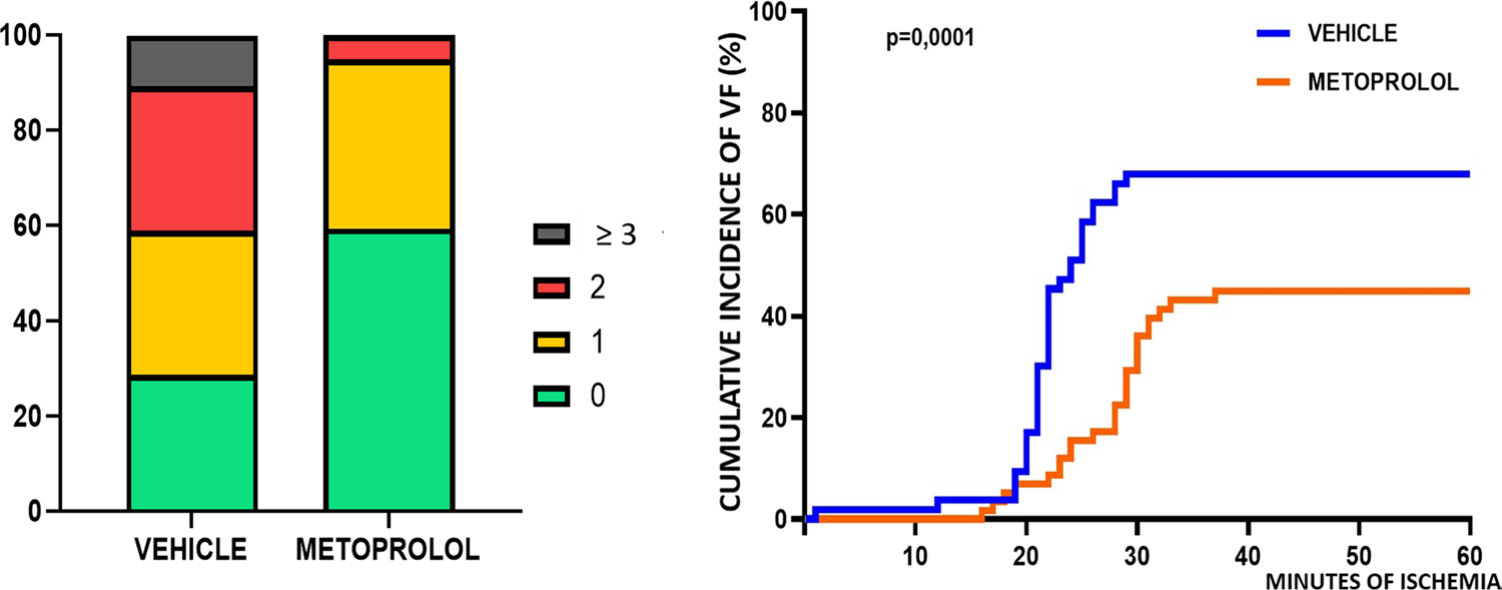
Incidence and timing of primary ventricular fibrillation. Left panel: percentage of animals with ventricular fibrillation (VF) per group (pooled data). The color scale ranking highlights the difference in VF incidence between pigs receiving saline and pigs receiving metoprolol. Right panel: plot of cumulative VF incidence (percentage of animal suffering at least one VF) against time from ischemia onset to first VF

Experimental MI was induced in 122 pigs. Of these, two animals died during MI induction due to refractory VF (1 in a metoprolol group 1 in a vehicle group). A further 4 animals died during the week after MI induction, before completing the 7-day CMR (1 in a metoprolol group, 3 in vehicle groups), and 14 animals died between day 7 and day 45, thus only undergoing the day 7 CMR (4 in metoprolol groups, 10 in vehicle groups). Therefore, overall mortality was 26.2% among vehicle-treated pigs vs. 9.8% among metoprolol-treated pigs (*p* = 0.07) (Fig. 2). There were no differences in hemodynamic parameters across treatment allocation for any ischemia duration (Table 1). Of note, the incidence and number of VF episodes during ongoing ischemia were significantly lower in animals allocated to metoprolol (36.5% for vehicle vs. 20.8% for metoprolol; *p* = 0.001) (Fig. 3).

**Table 1:** Hemodynamic parameters during myocardial infarction induction

	Vehicle	Metoprolol	*p* value
Baseline (before myocardial infarction induction)			
Heart rate (beats per min)	76.6 ± 9.5	76.2 ± 9.9	0.860
Systolic blood pressure (mmHg)	113.1 ± 8.0	113.5 ± 7.8	0.821
Diastolic blood pressure (mmHg)	71.5 ± 5.8	69.1 ± 7.1	0.079
Post-reperfusion			
Heart rate (beats per min)	91.8 ± 9.6	90.9 ± 7.8	0.612
Systolic blood pressure (mmHg)	88.8 ± 9.6	87.5 ± 9.9	0.498
Diastolic blood pressure (mmHg)	58.8 ± 6.3	56.8 ± 6.1	0.125

Data from animals in different ischemia duration protocols were pooled according to treatment allocation (metoprolol or vehicle)

Data are presented as mean ± standard deviation unless otherwise noted

### Infarct size trajectories over time in the pig infarction–reperfusion model

**Fig. 4:**
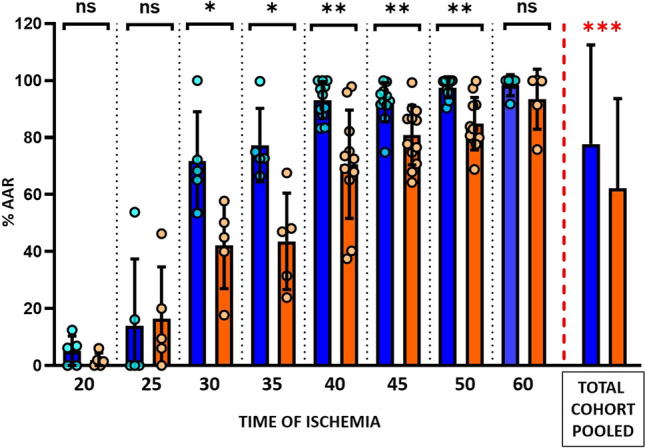
Infarct size (normalized to area at risk) according to the duration of ischemia in metoprolol and vehicle-treated pigs. Infarct size as % of the AAR (area at risk) at 7-day CMR follow-up in groups allocated to different lengths of ischemia. Blue columns represent vehicle groups; orange columns represent metoprolol groups. Pooled total cohorts represent animals receiving vehicle (blue) versus animals receiving metoprolol (orange) irrespective of ischemia duration. Data are presented as median and IQR. Dots represent data for individual animals. *ns* non-significant; *p < 0.05, **p < 0.01, ***p < 0.001

**Fig. 5:**
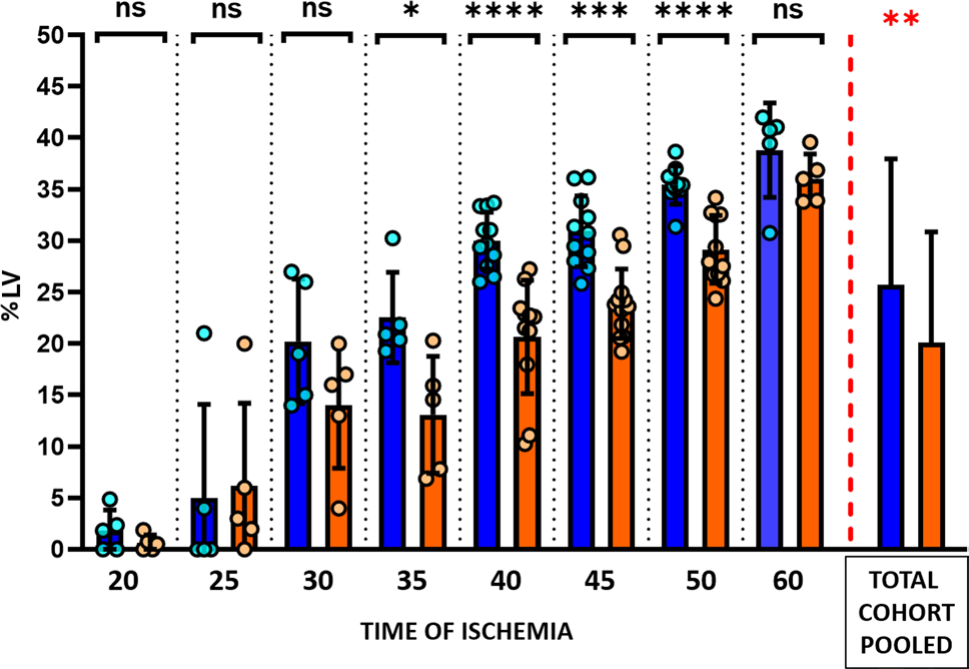
Absolute infarct size according to duration of ischemia in metoprolol and vehicle-treated pigs. Infarct size as % of the left ventricle at 7-day CMR follow-up in groups allocated to different lengths of ischemia. Blue columns represent vehicle groups; orange columns represent metoprolol groups. Pooled total cohorts represent animals receiving vehicle (blue) versus animals receiving metoprolol (orange) irrespective of ischemia duration. Data are presented as median and IQR. Dots represent data for individual animals. *ns* non-significant; *p < 0.05, **p < 0.01, ***p < 0.001, ****p < 0.0001. *LV* left ventricle

Infarct size was evaluated on day 7 CMR. Among pigs allocated to vehicle, infarctions in the 20-min I/R protocol were very small (6.2 [0.0–9.7] % AAR). Increases in ischemia duration from 20 to 35 min resulted is an exponential increase in IS (72.7 [69.0–87.3] %AAR). An ischemia duration of 40 min resulted in a further increase in IS to occupy almost the entire AAR (97.8 [87.9–100.0] % AAR) (Fig. 4). Absolute infarct size (% LV) followed the same temporal progression pattern (Fig. 5).

### Impact of early i.v. metoprolol administration on infarct size trajectories

**Fig. 6:**
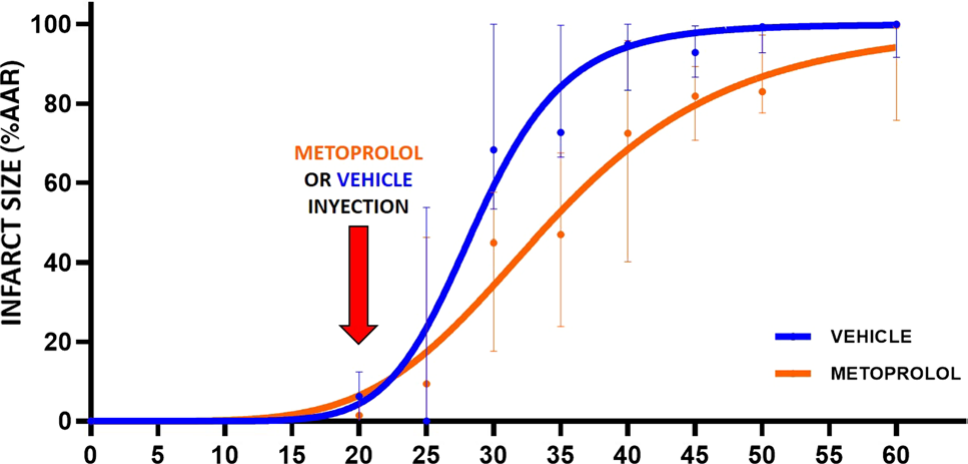
Time-dependent progression of infarct size in the presence or absence of metoprolol. Slope progression of infarct size (% AAR) with time of ischemia (minutes). Blue, vehicle group; orange, metoprolol group. Data are presented means (dots) ± SD (lines)

For all ischemia durations, i.v. metoprolol was injected 20 min after ischemia onset in animals allocated to active treatment. In the 20 and 25-min I/R groups, IS after metoprolol injection at min 20 did not differ significantly from that in vehicle-treated pigs (Figs. 4, 5). Infarcts in animals injected with metoprolol at min 20 and reperfusion at 30, 35, 40, or 50 min after ischemia onset were consistently smaller than in vehicle-treated counterparts (Figs. 4, 5). The protection afforded by metoprolol was lost when reperfusion was delayed to 60 min; pigs receiving metoprolol at min 20 and reperfused at 60 min had virtually the same infarct size as their vehicle-treated counterparts. IS trajectories in vehicle- and metoprolol-treated pigs are shown in Fig. 6.

Pooling of data from all ischemia duration protocols revealed that pigs receiving i.v. metoprolol had significantly smaller relative infarctions (% AAR) (Fig. 4) and absolute infarctions (% LV) than those injected with vehicle (Fig. 5).

### Attenuation of infarct size progression by early i.v. metoprolol administration

**Fig. 7:**
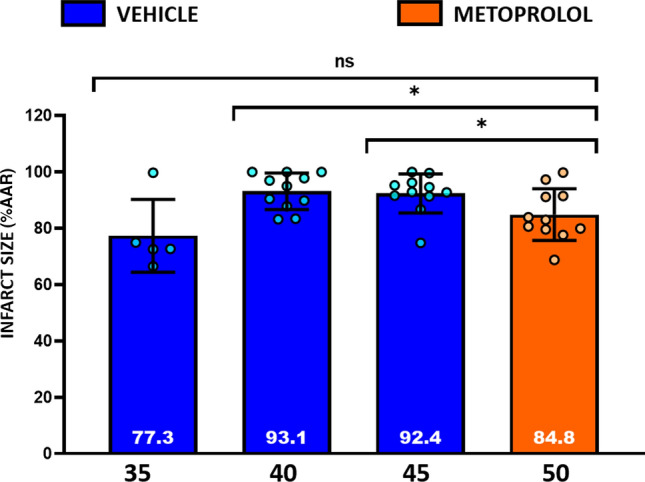
Early administration of metoprolol delays infarct progression. Differences in IS (% AAR) between animals receiving i.v. metoprolol and 50 min of ischemia and animals receiving i.v. vehicle and 35-, 40-, or 45-min ischemia. *ns* non-significant; *p < 0.05, **p < 0.01, ***p < 0.001, ****p < 0.0001

To evaluate the ischemic injury attenuation induced by early i.v. metoprolol injection in the course of an infarction, we compared IS in pigs undergoing 50-min I/R in the presence of metoprolol vs. those undergoing shorter I/R protocols without metoprolol. Infarct size (%AAR) in pigs undergoing 50-min I/R and injected i.v. with metoprolol at ischemia min 20 had significantly smaller infarctions than vehicle-treated pigs undergoing 40 and 45-min I/R protocols. IS (%AAR) in metoprolol-treated pigs undergoing 50-min I/R did not differ from that in control pigs undergoing 35-min I/R (Fig. 7).

### Impact of early i.v. metoprolol administration on left ventricular ejection fraction and microvascular obstruction

**Fig. 8:**
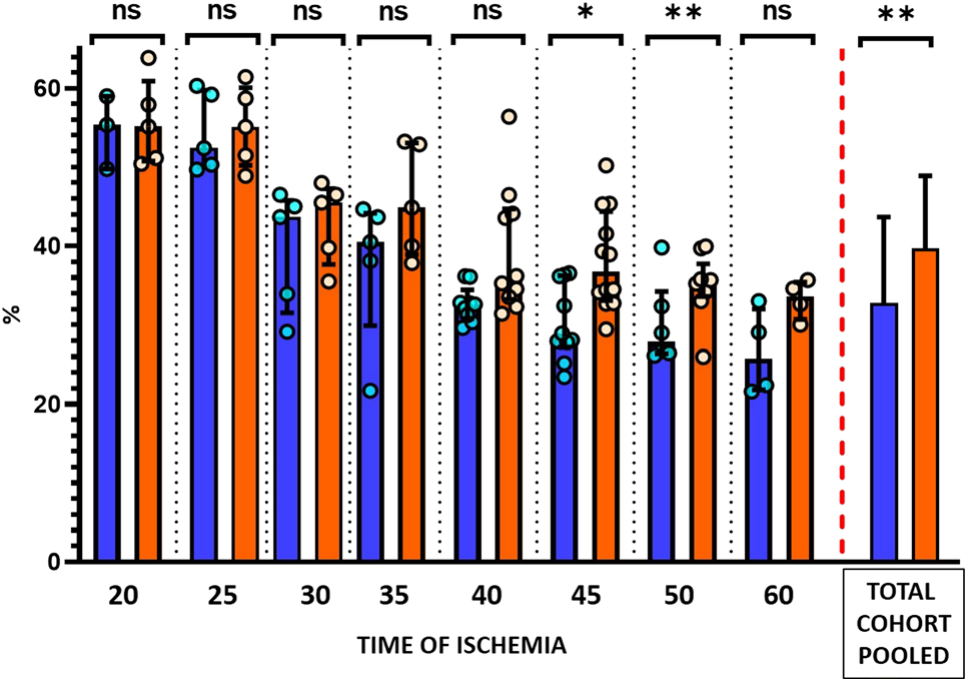
Long-term left ventricular ejection fraction according to duration of ischemia in metoprolol and vehicle-treated pigs. LVEF at 45-day CMR follow-up in groups allocated to different lengths of ischemia. Blue, vehicle groups; orange, metoprolol groups. Pooled total cohorts represent animals receiving vehicle (blue) versus animals receiving metoprolol (orange) irrespective of ischemia duration. Data are presented as Median and IQR. Dots represent data for individual animals. *ns* non-significant; *p < 0.05, **p < 0.01

Long-term (day 45) LVEF was higher in metoprolol-treated pigs in all ischemia duration protocols, although statistical significance was reached only in the 35- and 40-min I/R groups. Pooling of data from all ischemia duration protocols revealed that pigs receiving i.v. metoprolol had significantly higher long-term LVEF than those injected with vehicle (Fig. 8). MVO g) on day 7 CMR did not differ between vehicle- and metoprolol-treated pigs. All CMR parameters on day 7 and day 45 are shown for all ischemia duration protocols in Table 2.

**Table 2:** Cardiac magnetic resonance-derived parameters at day 7 and day 45 follow-up

	20-Min I/R			25-Min I/R			30-Min I/R			35-Min I/R			40-Min I/R		
	Vehicle	Metopr	*p* value	Vehicle	Metopr	*p* value	Vehicle	Metopr	*p* value	Vehicle	Metopr	*p* value	Vehicle	Metopr	*p* value
MDCT															
*N*	5	5		5	5		5	5		5	5		12	12	
AAR (% LV)	30.9 [28–36]	33.4 [28–38]	0.73	26.9 [23–35]	31.7 [29–38]	0.22	26.2 [22–32]	26 [24–30]	0.84	30 [28–32]	32 [30–33]	0.06	32.7 [29–34]	28.5 [27–31]	0.06
Day 7 CMR															
*N*	5	5		5	5		5	5		5	5		11	11	
IS (% AAR)	6.2 [0–10]	1.4 [0–4]	0.4	0 [0–35]	9.4 [3–33]	0.55	68.3 [59–86]	45 [28–54]	0.01	72.7 [70–87]	45 [28–58]	0.01	95 [88–100]	72 [65–82]	0.001
IS (% LV)	1.8 [0–4]	0.5 [0–1]	0.5	0 [0–12]	3 [1–13]	0.6	19 [14–26]	16 [8–18]	0.3	21 [19–26]	14 [7–18]	0.01	29.6 [27–33]	22.6 [18–23]	> 0.001
MVO (g)	0 [0–0]	0 [0–0]	0.9	0 [0–0]	0 [0–0]	0.9	0 [0–0]	0 [0–0]	0.9	0 [0–2]	0 [0–0]	0.9	0.1 [0–1]	0 [0–0]	0.2
Day 45 CMR															
*N*	3	5		5	5		5	5		5	5		9	10	
LVEF (%)	55.3 [49–59]	55.1 [50.78]	0.9	52.4 [50–59]	55.1 [50–60]	0.9	43.6 [31–45]	45.5 [38–47]	0.4	40.4 [30–44]	44.9 [39–53]	0.3	32.6 [31–34]	35.7 [33–45]	0.02

	45-Min I/R			50-Min I/R			60-Min I/R			Pooled cohort		
	Vehicle	Metopr	*p* value	Vehicle	Metopr	*p* value	Vehicle	Metopr	*p* value	Vehicle	Metopr	*p* value
MDCT												
*N*	12	12		12	12		5	5		61	61	
AAR (% LV)	31.5 [29–36]	31 [29–33]	0.48	35.7 [32–38]	35.1 [31–38]	0.55	38.2 [33–41]	39.4 [35–42]	0.7	31.9 [29–36]	31.3 [28–35]	0.8
Day 7 CMR												
*N*	11	12		10	11		5	5		57	59	
IS (% AAR)	93 [91–96]	82 [72–89]	0.002	99.4 [95–100]	83 [78–91]	0.001	100 [96–100]	99.8 [84–100]	0.3	91.7 [70–99]	72.5 [40–86]	< 0.001
IS (% LV)	30.7 [28–34]	23.7 [21–25]	< 0.001	35.4 [34–36]	28 [26–32]	< 0.001	40.7 [35–41]	36 [33–38]	0.22	29.5 [20–34]	22.6 [13–27]	0.001
MVO (g)	1.6 [1–3]	0.6 [0–1]	0.01	0.4 [0–4]	0 [0–1]	0.06	0.6 [0–1]	3 [0–4]	0.9	0 [0–1]	0 [0–0]	0.09
Day 45 CMR												
*N*	10	12		6	9		4	4		45	51	
LVEF (%)	28.5 [27–36]	36.7 [33–44]	0.005	27.8 [26–34]	35.2 [33–37]	0.1	25.7 [21–32]	33.6 [31–35]	0.1	32.8 [29–43]	40 [34–49]	0.001

Data are shown as median [interquartile range]

*LVEF* left ventricular ejection fraction, *MAR* myocardial area at risk, *metopr* metoprolol, *MVO* microvascular obstruction

## Discussion

In the present study, we used a translational pig I/R model to determine the trajectories of infarct size progression in control conditions and in the presence of i.v. metoprolol delivered 20 min after ischemia onset. Reperfusion was initiated at different timings after ischemia onset, ranging from 20 min (immediately after vehicle/metoprolol injection) to 60 min (40 min after vehicle/metoprolol injection). CMR was performed on day 7 to quantify the extent of irreversible injury and on day 45 to measure long-term LVEF. The principal finding of the present study is that i.v. metoprolol significantly delays infarction progression (Fig. 6). These data identify i.v. metoprolol as one of the few interventions that can be initiated after ischemia onset able to reduce the progression of irreversible injury. To date, only reperfusion itself [28], and remote ischemic per-conditioning [24], were demonstrated to have this anti-ischemic damaging effect.

In STEMI survivors, the extent of irreversible injury (myocardial IS) is a strong predictor of long-term adverse events [20, 35]. Consequently, the extent of CMR-LGE (a surrogate for IS) is recommended as the primary outcome in clinical trials and large animal studies addressing the effect of cardioprotective strategies during STEMI [19]. Due to their independent association with outcomes, LVEF and MVO are also recommended as secondary outcomes [19]. In the present study, we measured these three outcomes to evaluate the impact of metoprolol on irreversible myocardial damage. The beneficial effect of i.v. metoprolol injected during ischemia on all three surrogate markers strongly supports the potential of this intervention. It should be noted that despite metoprolol reduced MVO in some ischemia duration protocols, the incidence of this event was low in all preparations. It is well known that MVO is very frequent in different I/R models, including patients [12].

Classical studies using a large animal model of MI demonstrated that IS increases with time [28]. Other variables influencing the rate of irreversible injury progression include temperature and the presence of collaterals. Time-dependent IS progression was originally demonstrated in the dog [28], whose well-developed net of collaterals slows IS progression. Due to its anatomical and physiological similarities to humans, the large white pig is the most widely used I/R model [19]. Pigs lack collaterals, and IS progression is thus faster than in dogs. Our data show that most of the area subtended to ischemia becomes irreversibly injured between 20 and 35 min after ischemia onset. These data have important implications for future studies using this model because they show that, in the absence of strategies to alter ischemic injury, there is a very narrow time-window for reducing IS. Fast IS progression in humans usually occurs in young patients with no pre-existing collaterals, in whom large infarctions can be observed despite moderate ischemia durations [2]. This population is at much higher risk than older patients, who better tolerate prolonged ischemia. Time-dependent IS progression is generally slower in the clinical setting than in the pig model used here, with IS progression taking at least 2 h to occupy most of the AAR [8]. We, therefore, hypothesize that the timing of metoprolol injection in our study (20 min after ischemia onset, or half the time taken for IS to represent most of the AAR in the pig) might be comparable to the clinical setting of a patient presenting during the first hour of STEMI (half the time taken for IS to represent most of the AAR in humans).

Interventions able to reduce IS beyond the effect of reperfusion itself would have major prognostic implications, and as such their identification is an unmet clinical need [21]. In the past, most tested interventions have targeted reperfusion injury, under the premise that this form of damage significantly contributes to final IS [13, 15]. Unfortunately, most clinical trials of these interventions have failed to demonstrate a clinical benefit [10, 16, 36]. There is, therefore, renewed interest in identifying strategies able to delay the IS progression (ischemic injury) [31]. In the present study, we show that metoprolol significantly attenuates the time-dependent progression of IS (Fig. 6), thus positioning this strategy as one of few able to improve long-term outcomes in STEMI patients when applied in conjunction with reperfusion. Another intervention that has been shown to reduce ischemic injury is remote ischemic pre-conditioning. Kleinbongard et al. recently demonstrated in a similar pig model of reperfused MI that remote ischemic conditioning initiated after coronary occlusion resulted in attenuation of ST-segment elevation despite ongoing coronary occlusion and this translated into smaller MI size [24]. These data strongly suggest that this intervention attenuated ischemic injury. In the clinical setting, remote ischemic per-conditioning has recently failed to improve clinical outcomes in STEMI patients [11]. However, it should be noted that patients enrolled in this trial in many centers were enrolled in the cath lab, and the time from conditioning onset to reperfusion was very short many times, leaving a very small room for ischemia progression attenuation.

The infarct-limiting effects of metoprolol in STEMI patients undergoing PPCI have been tested in two randomized clinical trials (the METOCARD-CNIC [22, 27] and the EARLY-BAMI trials [29]), which produced different outcomes [26]. In the METOCARD-CNIC trial, metoprolol administration was associated with smaller infarcts [22] and higher long-term LVEF [27], whereas in the EARLY-BAMI study, metoprolol did not improve clinical outcomes [29]. In both trials, metoprolol was shown to be safe and to reduce the incidence of primary VF. An important difference between the trials is the timing of metoprolol administration. In METOCARD-CNIC, metoprolol was injected immediately after STEMI diagnosis, whereas in the EARLY-BAMI trial, some patients did not receive the full metoprolol dose, or received it when they reached the catheterization lab [26]. Our results reconcile the apparent contradictory trial findings and confirm that metoprolol most likely exerts its cardioprotective effect by slowing IS progression. Metoprolol is a potent protector against reperfusion injury in mouse models of I/R [6]; however, reperfusion injury appears to contribute less to final IS in pigs and humans than in rodents, and this difference might partially explain the failures to translate promising cardioprotective strategies to the clinic. In this regard, it has been proposed that there is a narrow ischemia duration window at which reperfusion-related injury significantly contributes to IS in large animals and humans [14]. If this was the case, part of the benefits of metoprolol seen in our study could have also been the result of reperfusion-injury amelioration, which was seen at ischemia duration protocols of 30–50 min, neither shorter nor longer than this window. In this regard, we previously demonstrated that part of the metoprolol-related infarct-limiting effects was driven by a direct effect on neutrophils and neutrophil–platelet interactions, reducing reperfusion-related injury [6]. If this was also a prevailing mechanism in the large animal model, it might be very well the case that to induce neutrophil stunning in a significant number of cells, metoprolol needs a time of circulation. This could partially explain the benefits observed only when metoprolol was on board 10 min or more.

One aspect than deserves explanation is the lack of differences between metoprolol and vehicle in terms of heart rate or blood pressure at reperfusion. Metoprolol has a known hemodynamic effect not observed in our experimental setting. The reason for this is the use of continuous amiodarone infusion (300 mg/h) in both treatment groups. Amiodarone has strong effects on heart rate and blood pressure. It is noteworthy that even in the absence of differences in heart rate, metoprolol administration was associated with significant IS-reducing effect. These data clearly dissociate the heart rate reducing effect to the cardioprotective one and explain why other drugs that lower heart rate, such as calcium channel blockers, are not associated with IS-reducing effect.

Reperfusion (either mechanical by PPCI or pharmacological by fibrinolysis) has become the mainstay treatment for STEMI patients. Head-to-head comparison of these approaches clearly favors PPCI, which results in a more complete reperfusion with less MVO and is associated with fewer complications. However, PPCI is not always an immediate option, and triaging patients for PPCI delays reperfusion. Current guidelines recommend transfer of patients to the PPCI center over immediate fibrinolysis so long as this can be done within 120 min of STEMI diagnosis [21]. The significant slowing of IS progression afforded by metoprolol could be especially beneficial for patients facing a long journey time to the PPCI center. Early administration of metoprolol, especially to patients presenting anterior infarctions, early in the course of STEMI (within 1–2 h from symptom onset), might extend the time window for selecting PPCI over fibrinolysis, thus allowing the best reperfusion strategy to be offered to patients who otherwise would receive fibrinolysis. As a limitation to this hypothesis the effects of metoprolol added to fibrinolytic therapy is unknown.

An additional benefit of metoprolol is the reduction in VF, shown here and in previous clinical trials [26]. In this regard, it has been shown in the pig model of reperfused MI that the incidence of VF (not the number of events or defibrillations) is associated with larger IS [34]. Skyschally et al. showed that VF is associated with larger AAR and lower residual myocardial blood flow [34]. Given that in our study AAR was not different between groups, it is highly plausible that metoprolol had an impact on residual blood myocardial flow and this resulted in a reduced incidence of VF, as well as in a reduction of the ischemic injury progression. Clinical practice guidelines indicate i.v. beta-blockers upon STEMI diagnosis with a class of recommendation IIa and level of evidence A [21]. However, this recommendation is rarely implemented [9]. This seems a lost opportunity for STEMI patients given the strong attenuation of time-dependent IS progression shown here for metoprolol, plus its safety when administered to stable patients (Killip class I–II) and its potency in reducing primary VF [22, 29]. What is needed is a definitive randomized clinical trial enrolling patients presenting far from the PPCI center (e.g. at the limit of the 120 min delay) to demonstrate the clinical benefits of early i.v. metoprolol administration and thus convince the clinical community of the value of this therapeutic strategy.

### Limitations

Our conclusions are limited by the use of the pig model. Pigs are widely used as a translational model because of the similarities of their hemodynamic parameters and heart and coronary anatomy to humans [4, 19]. However, the pig heart has poor collateral flow, and this lack of collaterals may translate into a faster progression of myocardial damage than occurs in patients. Other potential limitation is referred to VF, interactions between metoprolol and amiodarone could affect this anti-arrhythmic effects.

## Conclusions

Using a pig model of reperfused STEMI, we have shown that early i.v. injection of metoprolol is able to delay the time-dependent progression of irreversible myocardial damage. IS in pigs receiving metoprolol at 20-min ischemia and reperfused at 50 min was equal to that of vehicle-treated pigs undergoing ischemia for 35 min (30% less ischemia time) and significantly smaller than vehicle-treated pigs reperfused at 40 min. Early i.v. metoprolol injection in the course of experimental MI results in better long-term LVEF. Metoprolol significantly reduced the incidence of primary VF during ischemia and long-term mortality. Early i.v. metoprolol administration seems best suited for patients presenting early in the course of STEMI with a long anticipated transport time to the PPCI center.
